# Impact of Androgen Deprivation on Oxidative Stress and Antioxidant Status in Nigerian Patients With Prostate Cancer Undergoing Androgen Deprivation Therapy

**DOI:** 10.1200/GO.20.00290

**Published:** 2020-10-01

**Authors:** Iya Eze Bassey, Bukola Abosede Emodi, Uwem Okon Akpan, Idorenyin Francesca Akpan Iyakndue, Edim Azubuike Anakebe, Bassey Edward Icha, Henry Afamuefuna Efobi, Akan Joshua Ntinya, Alphonsus Ekpe Udoh

**Affiliations:** ^1^ Medical Laboratory Sciences Department, Faculty of Allied Medical Sciences, College of Medical Sciences, University of Calabar, Calabar, Nigeria; ^2^ Department of Chemical Pathology, University of Calabar Teaching Hospital, Calabar, Nigeria

## Abstract

**PURPOSE:**

Prostate cancer (CaP) incidence and mortality rate are increasing in Africa. Some have linked oxidative stress with the pathogenesis of cancer. This study assessed the levels of malondialdehyde (MDA), nitric oxide (NO), total plasma peroxide (TPP), and total antioxidant capacity (TAC) in Nigerian patients with CaP.

**PATIENTS AND METHODS:**

One hundred twenty patients with CaP and 100 apparently healthy controls were consecutively recruited into this case-control study. The patients with CaP were divided into treatment-naïve and androgen deprivation therapy (ADT)–treated groups. Anthropometric indices were measured, and MDA, NO, TAC, and TPP were assayed by colorimetric methods. The *t* test and analysis of variance were used in analysis of data; statistical significance was set at *P* < .05, and 95% CIs were reported.

**RESULTS:**

The patients with CaP had significantly higher waist-hip ratios and NO (*P* = .0001), TPP (*P* = .001), oxidative stress index (OSI; *P* = .003), and MDA values (*P* = .002) than controls. The treatment-naive patients with CaP had significantly higher waist-hip ratios (*P* = .011) and TPP (*P* = .013), MDA (*P* = .011), and NO values (*P* = .0001) and lower TAC values (*P* = .013) compared with the controls. The ADT-treated patients had higher waist-hip ratios (*P* = .0001) and TPP (*P* = .005), OSI (*P* = .005), MDA (*P* = .011), and NO values (*P* = .0001) than the controls. However, the treatment-naive patients had significantly higher NO values (*P* = .05) only compared with the ADT-treated patients. There was a significantly positive correlation between MDA and duration of treatment (*r* = 0.280, *P* = .018) in ADT-treated patients with CaP.

**CONCLUSION:**

This study demonstrated that patients with CaP have higher levels of TPP, MDA, and NO and lower levels of TAC compared with men without CaP. In addition, even in patients with CaP undergoing treatment, TPP and MDA levels remained high.

## INTRODUCTION

Prostate cancer (CaP) has now become a global health problem.^1^ CaP is the most significant cancer that has disproportionately overburdened men of African ancestry.^2^ The most prevalent cancer in Nigerian men is CaP; it constitutes approximately 6.1%-19.5% of all cancers in Nigeria, and the number of men being diagnosed with CaP is increasing,^3^ with an average prevalence of 11%.^4^

**Context**

**Key Objective**
What is the pattern of changes in oxidative stress markers in native black men with prostate cancer (CaP) undergoing androgen deprivation therapy?
**Knowledge Generated**
Oxidative stress is increased and antioxidant status decreased in patients with CaP, as shown by higher total plasma peroxide, malondialdehyde, and nitric oxide levels and lower total antioxidant capacity compared with men without CaP. Even in patients undergoing androgen deprivation therapy, total plasma peroxide and malondialdehyde levels remained high, and the levels of malondialdehyde also increased with duration of treatment.
**Relevance**
Elevated oxidative stress markers are independently associated with poorer outcomes. Therefore, this should be addressed in the management of patients with CaP.

CaPs are solid tumors of glandular origin.^5^ Androgens are vital for the normal functioning, growth, and development of the prostate and have been linked to prostate carcinogenesis.^6^ CaP cells that thrive without androgens normally possess a phenotype that is aggressive. Although many signaling pathways and factors have been linked to aggressive CaP, the trigger for initiation and promotion of malignancy is still a topic of debate.^7^ Substantial evidence shows that both genetic and environmental influences have vital roles in the evolution of CaP.^8^

A significant amount of evidence suggests that oxidative stress and its cumulative impact on DNA damage are key aging-associated influences on prostate carcinogenesis.^9^ Oxidative stress is caused by an imbalance in reactive oxygen species (ROSs) and reactive nitrogen species (RNSs) production and the cell’s detoxification defense system, favoring an ROS-rich environment and/or reduced antioxidant reserves.^10^ Examples of ROSs and RNSs responsible for oxidative stress include superoxide, ozone, hydrogen peroxide, hydroxyl radical, nitrate, nitroxyl, and nitrosothiol.^11^ These ROSs are produced as by-products of metabolic processes in cells^12^ and play a vital role in regulating several biologic phenomena, some of which are associated with neoplastic transformation and abnormal growth.^13^ The extent of lipid peroxidation in the body is also important. It is a multifaceted process that includes lipid radical formation and propagation, oxygen uptake, double bond rearrangement of unsaturated lipids, and subsequent damage of membrane lipids, with the resultant production of ketones, alcohols, aldehydes, alkanes, and ethers.^14^ Lipid peroxidation typically causes a considerable amount of DNA-malondialdehyde (MDA) adducts. Measuring total plasma peroxides (TPP) and MDA is a way to estimate the extent of lipid peroxidation in the body.^14,15^

Enzymatic antioxidants and nonenzymatic antioxidants act together in detoxifying the effects of oxidative stress and lipid peroxidation.^16^ This synergistic action can be measured using total antioxidant capacity (TAC).^17^ Oxidative stress occurs as a result of an imbalance in ROS production and effectiveness of the defense by antioxidants. Products of lipid peroxidation and nonenzymatic and enzymatic antioxidant levels are used as oxidative stress markers.^18^

Nitric oxide (NO) is a ubiquitous free radical signaling molecule that regulates several cellular processes including apoptosis, smooth muscle tone, immune response, angiogenesis, and synapse communication.^19^ Despite its numerous physiologic functions, NO has also been linked to the etiology and progression of cancer and other diseases.^20^ NO’s role in cancer is complex and ranges from being an etiologic agent to being a curative agent. NO can be genotoxic under some circumstances, indicating its probable involvement in the etiology of many cancers.^19-21^

Most of the extensive experimental and clinical studies over the years have been carried out on White populations and African Americans. There is a scarcity of literature or published work on this subject in native black men. Investigation into all aspects of this disease is essential because the incidence of CaP is increasing almost geometrically in the African population, and the incidence in any locale is influenced by a number of factors peculiar to that environment. Therefore, this study assessed serum MDA, NO, TAC, TPP, and oxidative stress index (OSI) in patients with CaP in Nigeria.

## PATIENTS AND METHODS

### Study Design and Participant Selection

A case-control study design was used for this study. A total of 220 participants were consecutively recruited for the study. They were between 40 and 90 years old and were all Nigerians. One hundred twenty patients of Nigerian origin with CaP, who attended the Urological Clinic of the University of Calabar Teaching Hospital, Calabar, Cross River state, Nigeria, were recruited as test participants. One hundred apparently healthy participants without benign prostatic hyperplasia or CaP were also consecutively recruited as controls from places of worship, community events, markets, offices, and workplaces in Calabar metropolis, Cross River state. The patients with CaP were subgrouped as androgen deprivation therapy (ADT)–treated patients and ADT treatment–naive patients. ADT modalities included medical castration (gonadotropin-releasing hormone, bicalutamide, and flutamide) and surgical orchiectomy. The World Medical Association Declaration of Helsinki standards were observed in this study. Ethical clearance was obtained from University of Calabar Teaching Hospital Health and Research Ethical Committee (UCTH/HREC/33/543). The participants gave their written informed consent before being enrolled in the study. A standard questionnaire was used to obtain relevant information.

### Sample Size Calculation

Sample size and power calculations for this unmatched case-control study were done using the StatCalc function of Epi Info software (Centers for Disease Control and Prevention, Bethesda, MD). A two-sided confidence level of 95%, desired power of 80%, 1:1 ratio of controls to cases, and odds ratio of 2.5 were used. The percentage outcome in the unexposed group was 50%. The Fleiss with continuity correction equation^22^ gave a sample size of 90 each for cases and controls. Taking into account an attrition rate of 10%, the sample size increased to 99 per group (ie, a total of 198 participants).

### Inclusion and Exclusion Criteria

The participants were older than age 40 years. Men with benign prostatic hyperplasia and men who were smokers were excluded. Controls with prostate-specific antigen (PSA) levels > 4 ng/mL were also excluded.

### Sample Collection

Venous blood (5 mL) was aseptically collected from each participant by venipuncture, allowed to clot, and spun in a centrifuge at 3,000 rpm for 5 minutes, and serum was obtained. Serum was stored in aliquots at −20°C until analyzed.

### Assay Methods

Blood pressure and anthropometric indices were measured as described by Akpan and Bassey.^23^ Body mass index (BMI) was computed as the ratio of weight (kilograms) to the square of height (meters), and waist-to-hip ratio was defined as waist girth/hip circumference.

#### PSA.

An enzyme-linked immunosorbent assay kit (Syntron Bioresearch, Carlsbad, CA) was used to determine PSA levels.

#### TAC.

The method of Koracevic et al^24^ was used to assess TAC. In this method, H_2_O_2_ reacts with a standard solution of Fe-EDTA complex by a Fenton-type reaction, which leads to hydroxyl radical formation. The OH^−^ formed then degrades benzoate, causing the release of thiobarbituric acid reactive substances (TBARS). Antioxidants present in the sample added causes suppression of TBARS production. This was read at 532 nm. Inhibition of development of color defined the TAC of the sample.

#### TPP.

TPP was determined according to the ferrous oxidation–xylenol orange (FOX)-2 method,^25^ with minor modifications.^26^ TPP was assayed by mixing 200 µL of serum with 1.8 mL of FOX-2 reagent in a glass tube and incubated for 30 minutes at room temperature. Standard used was 0.5 mmol/L H_2_O_2_ and was treated as the test. The mixture was centrifuged for 15 minutes at 3,000 rpm, and supernatant was extracted. Absorbance of the supernatant was read at 560 nm.

#### MDA.

The method of Buege and Aust^27^ was used for estimation of MDA. MDA was assayed by adding 100 µL of serum to 1.25 mL of 10% trichloroacetic acid, incubated at 90°C for 15 minutes. The mixture was then allowed to cool and centrifuged at 4,000 rpm for 10 minutes. The supernatant was extracted, added to 0.5 mL of 0.675% thiobarbituric acid, and incubated for 3 minutes. Absorbance was read at 532 nm.

#### NO.

The Griess assay method of Ghasemi and Zahediasl^28^ was used for NO. This assay assesses total levels of the metabolites NO_2_^−^ and NO_3_^−^ in samples. Zinc sulfates were used to deproteinize serum samples, and the samples were centrifuged for 10 minutes at 10,000 *g* and the supernatant extracted for analysis. Any NO3^−^ present in the supernatant was reduced to NO_2_^−^ by the action of vanadium (III) chloride. Griess reagent was then added and read at 540 nm. A standard curve of known nitrite concentrations was prepared, and sample concentrations were extrapolated from it.

#### OSI.

OSI (an indicator of the degree of oxidative stress) was calculated as the ratio of TPP to TAC.^26^

### Statistical analysis

Data were analyzed by SPSS software version 18 (SPSS, Chicago, IL). The results were expressed as means and standard deviations. The *t* test and one-way analysis of variance were used to compare data between and among groups, respectively, and least significant difference was used for post hoc analysis. Variables were correlated by Pearson correlation. The level of significance was set at 95% CI, where a probability value of *P* < .05 was considered statistically significant.

## RESULTS

The sociodemographic characteristics of the study participants are listed in Table 1. The Ekoi tribe contributed the greatest number of patients with CaP (25.8%), whereas the Yoruba tribe contributed both the fewest patients with CaP and the fewest controls. The highest participating tribe in the control group was the Ibibios tribe (31%). The majority of the participants were married in both the patient and control groups (90.8% and 90%, respectively). The majority of the patients with CaP (25.8%) were retired, whereas the majority of the controls were civil or public servants (32%). Only 15% of the patients with CaP had a smoking history compared with 9% of controls, and 10% of patients with CaP had a family history of CaP.

**TABLE 1:**
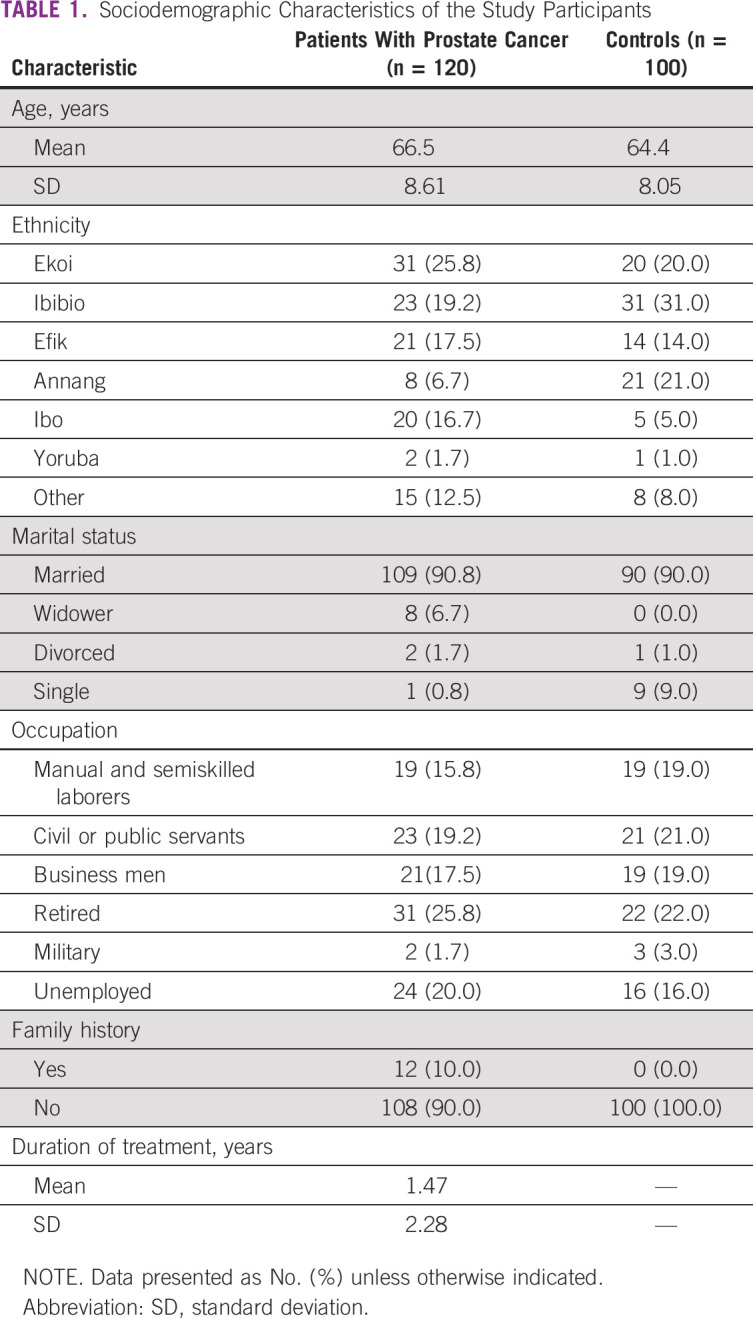
Sociodemographic Characteristics of the Study Participants

The patients with CaP had a significantly higher PSA, waist-hip ratio, NO (*P* = .0001), TPP (*P* = .001), OSI (*P* = .003), and MDA (*P* = .002) compared with the controls. However, there were no significant differences (*P* > .05) in the mean values of the other parameters between the two groups (Table 2).

**TABLE 2:**
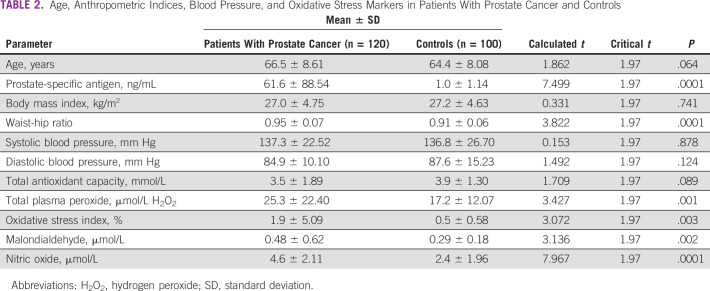
Age, Anthropometric Indices, Blood Pressure, and Oxidative Stress Markers in Patients With Prostate Cancer and Controls

**FIG 1:**
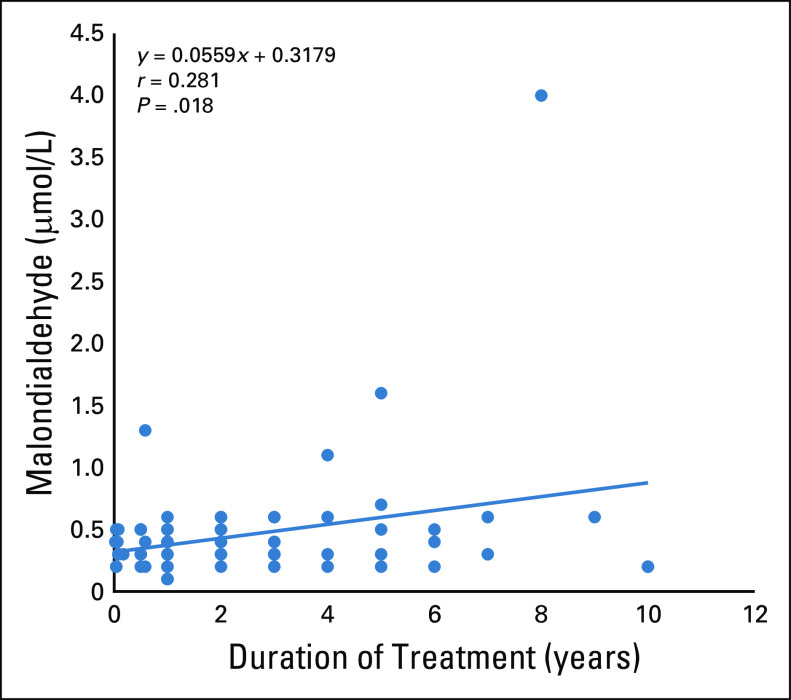
Correlation between malondialdehyde and duration of treatment in men undergoing androgen deprivation therapy.

There was a significant variation in the mean values of PSA (*P* = .0001), waist-hip ratio (*P* = .0001), TAC (*P* = .041), TPP (*P* = .006), OSI (*P* = .011), MDA (*P* = .015), and NO (*P* = .0001). There were no significant variations (*P* > .05) in the mean values of the other parameters among the groups (Table 3). A post hoc analysis showed that the treatment-naïve patients with CaP had a significantly higher PSA (*P* = .0001), waist-hip ratio (*P* = .011), TPP (*P* = .013), MDA (*P* = .011), and NO (*P* = .0001) and lower TAC (*P* = .013) compared with the controls. The ADT-treated patients had higher PSA (*P* = .0001), waist-hip ratio (*P* = .0001), TPP (*P* = .005), OSI (*P* = .005), MDA (*P* = .011), and NO (*P* = .0001), but there was no significant difference (*P* > .05) in TAC levels compared with the controls. However, when the treatment-naive patients and the ADT-treated patients were compared, all of the aforementioned parameters were comparable except for PSA and NO, which were higher in treatment-naive patients compared with the ADT-treated patients (Table 4). There was a significantly positive correlation between MDA and duration of treatment (*r* = 0.280, *P* = .018) in the ADT-treated patients with CaP (Fig 1).

**TABLE 3:**
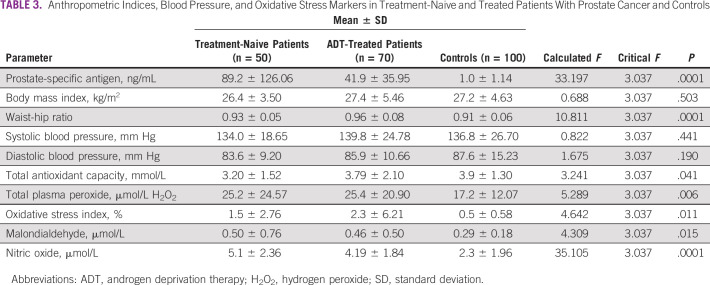
Anthropometric Indices, Blood Pressure, and Oxidative Stress Markers in Treatment-Naive and Treated Patients With Prostate Cancer and Controls

**TABLE 4:**
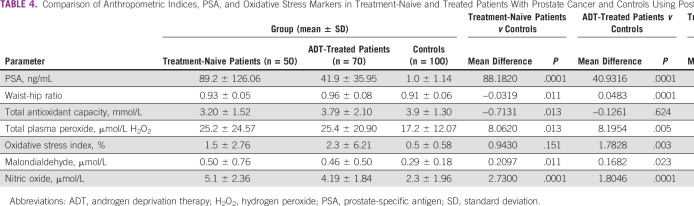
Comparison of Anthropometric Indices, PSA, and Oxidative Stress Markers in Treatment-Naive and Treated Patients With Prostate Cancer and Controls Using Post Hoc Analysis

## DISCUSSION

Over the past few decades, several studies have shown that oxidative stress markers are linked with the pathogenesis of cancer.^29^ In our study, it was observed that the treatment-naive patients with CaP had a significantly higher TPP, MDA, and NO and lower TAC compared with the controls, which points to increased oxidative stress. Oxidative stress can be caused by an increase in ROS levels and/or a depletion of cellular antioxidant capacity, most often over a prolonged period, which may result in genetic and epigenetic effects.^30^ Epigenetic events cause hypermethylation (via promoter) of GSTP1 and Nrf2, which reduces their expression and severely diminishes cellular antioxidative capacity. Excessive production of ROS (by metabolic alterations, extrinsic environmental factors such as inflammation, androgen receptor [AR] activation and mitochondrial dysfunction induced by mutations, and xenobiotic metabolism and hypoxia) is thus unopposed, favoring oxidative stress.^31^ NO also enhances cyclooxygenase activity in inflammatory cells in the interstitial spaces and epithelium of human prostate tissue.^32^

Oxidative stress can modify nuclear and mitochondrial DNA. ROS oxidizes DNA bases, leading to DNA hypermethylation and mutations. When these mutations are incorporated into genes that are involved in the control of the cell cycle, alterations in metabolic rate and/or cellular response take place. Consequently, mutations that occur in genes associated with cancer cause defects in the repair of DNA as well as cell cycle deregulation, which support tumorigenesis and development of CaP.^33^ ROSs also cause lipid peroxidation in membranes and thus alter their physicochemical properties. Unlike free radicals, these aldehydes can diffuse within the cell or even escape from it as a result of their relative stability, which enables them to attack targets distant from the original event site; thus, they become second cytotoxic messengers for the primary reactions.^34^ MDA is reportedly the most mutagenic as a result of its ability to form DNA adducts.^35^ In addition, ROSs cause carbonylation of proteins, leading to formation of reactive aldehydes and ketones in proteins that cause alterations in protein signaling and consequently modulation of cell signaling and activation of target genes that promote survival, progression, and metastasis of CaP.^31^ The upregulation of ERp57 by oxidative stress may have an important role in increasing both cell viability and resistance to stress. The transcription factors for some cancer survival proteins, such as PRDX6 and Hsp27, are increased by oxidative stress, thus protecting CaP cells from oxidative stress–induced necrosis.^36^ ROSs have also been shown to induce carcinogenesis through increase in the expression of STAMP2 via the *ATF4* gene. One of the effects of STAMP2 is the subsequent increase of ROS through its iron reductase activity, which helps to maintain a pro-oxidant milieu.^37^ This agrees with findings by Aydin et al^38^ and Akiibinu et al.^35^ However, Kucukdurmaz et al^39^ reported comparable MDA levels in patients with CaP and controls.

The ADT-treated patients with CaP similarly had a significantly higher TPP, MDA, and NO and a higher OSI compared with the controls. This also definitely suggests increased oxidative stress, even when undergoing ADT. The levels of the lipid peroxidation product (MDA) also increased with duration of treatment, as shown by the positive correlation between MDA and duration of treatment in the ADT-treated patients with CaP. ADT can trigger oxidative stress by causing the levels of ROS to increase and/or by decreasing cellular antioxidant capacity. Oxidative stress caused by ADT then increases AR activation by AR overexpression, de novo androgen synthesis, AR activation, expression of AR splice variants, mutation of AR and its related proteins, and alterations in non-AR signaling. These changes seem to support CaP cell survival despite ADT, subsequently leading to the development of castration-resistant CaP.^40^ This may develop within 2-5 years; even though the patient’s androgen levels are at castrate levels, the tumors keep proliferating and the patient is faced with a drug-resistant, lethal form of CaP.^41^

Although development of several cancers has been associated with obesity,^42^ worldwide studies are still controversial regarding the association between CaP incidence and BMI.^43-45^ One study reported no significant relationship between BMI and high-grade CaP detection but reported a significantly higher waist-hip ratio in patients with CaP compared with men without cancer.^45^ This report is similar to the findings of our study, which showed comparable BMI values but higher waist-hip ratio in both the treatment-naive and ADT-treated patients with CaP compared with controls. ADT has been shown in other studies to increase measures of central obesity independent of the type of ADT, and central obesity is one of the first metabolic changes to occur.^46,47^ This may be a result of the fact that testosterone deficiency caused by ADT results in decreased visceral adipose tissue lipolysis and accumulation of abdominal fat stores.^48^ Previous studies have shown a correlation between overproduction of ROS and fat accumulation, which may lead to lipid metabolic pathway dysfunction through direct lipid peroxidation, thereby maintaining a vicious cycle that, if not checked, may encourage CaP recurrence. This theory is supported by the positive correlation between duration of treatment and MDA observed in our study.

Although clinical trials in castration-resistant CaP have led to availability of some drugs for therapeutic use,^49^ there is always room to explore other avenues for therapy. An interesting angle to tackle oxidative stress may be through dietary antioxidant therapy. This is an area of future research that would be highly beneficial to Africans in general. A study that shows the major benefits of antioxidant therapy based on African foods would be beneficial because these foods would be readily accessible and available to these patients. In addition, the health benefits of nutrients and phytochemicals observed in trials and epidemiologic studies on humans consuming antioxidant compounds were predominantly seen when they were taken within their natural food matrices (eg, as vegetables, fruits, or grains). This is probably because of the generally low concentrations of nutrients and nonnutrients in these foods, as well as the synergistic actions of complex mixtures of nutrients and phytochemicals.^50^ In addition, lifestyle modifications that help control adiposity and perhaps the dyslipidemia associated with ADT may be other beneficial areas of research.

This study has shown that oxidative stress is increased and antioxidant status decreased in patients with CaP irrespective of treatment status and that MDA levels increased with duration of treatment. Because elevated oxidative stress markers are independently associated with poorer outcomes, this should be addressed in the management of patients with CaP. A limitation of this study was that experiments were not carried out to test the benefits, if any, of antioxidant therapy. Therefore, we cannot say whether antioxidant therapy may be beneficial to this group of patients.

This study showed that patients with CaP have higher levels of TPP, MDA, and NO and lower TAC compared with men without prostate cancer. In addition, even in patients with CaP undergoing treatment, TPP and MDA levels remained high.
